# Prediction of Strength Properties of Concrete Containing Waste Marble Aggregate and Stone Dust—Modeling and Optimization Using RSM

**DOI:** 10.3390/ma15228024

**Published:** 2022-11-14

**Authors:** Syed Roshan Zamir Hashmi, Muhammad Imran Khan, Shabir Hussain Khahro, Osama Zaid, Muhammad Shahid Siddique, Nur Izzi Md Yusoff

**Affiliations:** 1 Department of Structural Engineering, Risalpur Campus, National University of Sciences & Technology, Islamabad 44000, Pakistan; 2 Department of Transportation and Geotechnical Engineering, Risalpur Campus, National University of Sciences & Technology, Islamabad 44000, Pakistan; 3 Department of Engineering Management, College of Engineering, Prince Sultan University, Riyadh 11568, Saudi Arabia; 4 Department of Civil Engineering, Swedish College of Engineering and Technology, Wah 47080, Pakistan; 5 Department of Civil Engineering, Universiti Kebangsaan Malaysia, Bangi 43600, Selangor, Malaysia

**Keywords:** marble waste aggregates, stone dust, optimized mix, response surface methodology, environmentally friendly, sustainability, normal strength concrete

## Abstract

Carbon footprint reduction, recompense depletion of natural resources, as well as waste recycling are nowadays focused research directions to achieve sustainability without compromising the concrete strength parameters. Therefore, the purpose of the present study is to utilize different dosages of marble waste aggregates (MWA) and stone dust (SD) as a replacement for coarse and fine aggregate, respectively. The MWA with 10 to 30% coarse aggregate replacement and SD with 40 to 50% fine aggregate replacement were used to evaluate the physical properties (workability and absorption), durability (acid attack resistance), and strength properties (compressive, flexural, and tensile strength) of concrete. Moreover, statistical modeling was also performed using response surface methodology (RSM) to design the experiment, optimize the MWA and SD dosages, and finally validate the experimental results. Increasing MWA substitutions resulted in higher workability, lower absorption, and lower resistance to acid attack as compared with controlled concrete. However, reduced compressive strength, flexural strength, and tensile strength at 7-day and 28-day cured specimens were observed as compared to the controlled specimen. On the other hand, increasing SD content causes a reduction in workability, higher absorption, and lower resistance to acid attack compared with controlled concrete. Similarly, 7-day and 28-day compressive strength, flexural strength, and tensile strength of SD-substituted concrete showed improvement up to 50% replacement and a slight reduction at 60% replacement. However, the strength of SD substituted concrete is higher than controlled concrete. Quadratic models were suggested based on a higher coefficient of determination (R^2^) for all responses. Quadratic RSM models yielded R^2^ equaling 0.90 and 0.94 for compressive strength at 7 days and 28 days, respectively. Similarly, 0.94 and 0.96 for 7-day and 28-day flexural strength and 0.89 for tensile strength. The optimization performed through RSM indicates that 15% MWA and 50% SD yielded higher strength compared to all other mixtures. The predicted optimized data was validated experimentally with an error of less than 5%.

## 1. Introduction

Concrete has been a material of choice for construction purposes for ages [1]. The main ingredients of concrete, i.e., the binder (cement), fine aggregate (river sand), and coarse aggregate (crushed rock, gravel, etc.), remain the same with slight adaptation and modifications by the users. Owing to their economic availability and predictable behavior, natural aggregates account for the largest volume of solid minerals mined worldwide. About 47 to 50 billion tons of aggregate material are extracted annually worldwide, and the proportion of construction aggregates ranges from 68 to 85 percent [2]. Their alarming detrimental effects on the ecology and environment of the globe include damage to the basins of rivers, environmental pollution, and pH level variations in groundwater [3]. As an alternative to conventional concrete constituents, researchers have explored substitutions of these fundamental ingredients: cement, fine aggregate (sand), and coarse aggregate, with supplementary cementitious materials (SCM), as well as industry by-products and recycled materials. The substitutions chosen by researchers include blast furnace slag, quarry dust or crushed stone dust [4,5,6,7], silica-fume [8,9,10], rice-husk ash [11], marble slurry or powder [12,13,14,15,16,17,18], recycled aggregate from construction demolition waste [19,20,21,22,23,24], ornamental stone processing waste [25], coconut husk ash [10], steel fibers [11,26], sugarcane (bagasse) ash [27], nano or [22,28] carbon-fibers, graphene oxide powder [29], etc. in normal weight, self-compacting concrete [30] and high strength concrete, accordingly.

Correspondingly, the utilization of non-biological and non-degradable waste, such as that from the marble and stone crushing industries, the preeminent consumption led to research on the construction industry waste [31]. The huge quantity of discarded waste can be utilized in sustainable construction [32,33,34,35,36]. In examining the use of marble waste in concrete as an aggregate substitution, researchers found that concrete produced with 50% sand only, 75% aggregate only, or 25% both sand and aggregate replacements showed improved mechanical properties [12]. Researchers utilized various mixes using basalt, granite or limestone, and marble by replacing the primary aggregate at ratios of 20% to 100%. Marble aggregate showed low adhesion due to increased interface zone pores, a similar micro-structural matrix as standard reference concrete, and the same depth of carbonation, likely due to the lower alumina proportion of the coarse marble aggregate (CMA) mixes and a substantial rise in the migration coefficient of the chloride. The durability of CMA in environments with chloride contamination exhibited the poorest performance [17].

The use of waste from the marble industry and crushed concrete recycled aggregate (RA) in self-compacting concrete allows up to 100% replacement. The authors observed increased workability by using marble waste (MW) aggregate. They noted 6% & 5.6% lower compressive strengths at lower water-binder ratios and 31.5% & 7% at the ratio of water-binder as 0.4, respectively, but strain capability rises by using RA and MW aggregates as compared to LS stress-strain curves [23]. Researchers replaced natural aggregate with marble coarse aggregate in varying percentages from 0 percent as in the control sample to 100% (test specimen) by weight at a 0.6 w/c ratio in concrete mixes. The authors observed 14% more workable concrete in contrast to the control sample. When marble was used until 80% substitution of natural aggregate, an increase in average compressive strength of up to 40% at the initial 7-day and up to 18% at 28 days, respectively, was observed. They concluded that permeability was also increased due to the increased number of pores in the marble aggregate mix, whereas no significant variation in compressive strength reduction against acid exposure was observed [18]. Experimental investigation of marble dust and limestone powder concrete mix with 5, 10, and 15 percent substitutions of sand (fine aggregate), with cement content of 400 kg/ m^3^ (674 lb/yd^3^) and at a 0.5 w/c ratio. The authors concluded that the concrete mix with marble dust maintains greater compressive strength, higher resistance against sodium sulfate, and abrasion resistance, followed by limestone dust and least by the control specimen, respectively. They also established that water permeability was resisted by the specimen, with marble dust replacing 15% of it [37]. Singh, Srivastava, and Agarwal [38] concluded that once stone dust replaces the sand along with a 0.8% superplasticizer dose, the slump decreased from 84 mm (0% stone dust) to 0 mm (80% or more). Whereas when the dosage changed to maintain the workability, the slump varied from 60 mm at 0.6% (0% stone dust) to 35 mm at a 2.2% dose (100% stone dust), respectively. The 7-day compressive strength of concrete cubes was found to be at its maximum at 50% replacement as compared to all other percentages. Whereas, for 28 days, the maximum compressive strength of concrete cubes was established to be at 60% replacement as compared to all other percentages. Researchers have established that the compressive strength of mortar cubes at 28 days when combined in a ratio of (cement:{sand + stone powder}) 1:1 + 2. It would solve the dual purposes of economic construction as well as waste utilization for achieving sustainability [39]. 

In this study, marble waste aggregates (MWA) and stone dust (SD) from their respective productions were substantiated as an alternate approach. The source of MWA exists in the marble finishing industries. The estimated reserves of marble and onyx in Pakistan are more than three hundred billion tons. However, due to primitive ways of quarrying and subsequent processing, the marble processing units in Pakistan cause approximately 70% of the raw material to be transformed into solid and sludge waste [40]. Similarly, from quarries and stone crusher plants, stone dust, being the lowest size, is discarded too, piled up either outside processing plants or drained into water channels. This waste material from marble industries and crushing plants remains a source of air, water, and land pollution [19,31]. Similarly, as per the reports of the Small and Medium Enterprises Development Authority (SMEDA), Ministry of Industries & Production, Government of Pakistan, stone crushing is one of the prospective industries owing to the rising material demands in the construction and infrastructure sector in the country [41]. The current novel experimental study is unique from previous research in that it incorporates the WMA and SD into an optimum percentage without using any admixture in the concrete. The optimized substitutions of marble waste aggregate and stone dust were modeled with the help of RSM Model analysis as well as experimental confirmation. The specimens were subjected to physical, durability, and strength properties. The experimental and predicted results were compared and validated through analysis of variance (ANOVA) and statistical terms. The recycling of MWA and SD to replace natural aggregates would compensate for the unfavorable effects of each substitute material and produce the desired outcome in fresh and hardened concrete in a sustainable way. 

## 2. Materials and Methods

### 2.1. Materials

In the current study, marble waste aggregate (MWA) and stone dust (SD) were used as partial replacements for coarse aggregate and fine aggregate, respectively, for normal strength concrete. The conventional mix design had a mix ratio of 1:2:4 of cement: fine aggregate: coarse aggregate, respectively, and a water to cement ratio of 0.5. Ordinary Portland Cement (OPC) type-I was used and procured from the Cherat cement factory. The physical properties of OPC are given in Table 1, as determined according to ASTM C191-13, ASTM C187-11, and ASTM C430-08 standards [42,43,44]. 

Fine aggregate (medium coarse size sand) was obtained from the Lawrencepur Attack quarry and its physical properties are presented in Table 2. The coarse aggregate with a nominal maximum size of ¾ inch (20 mm) was the crushed stone of Margalla hill rock, and its physical properties are also shown in Table 2. 

**Figure 1:**
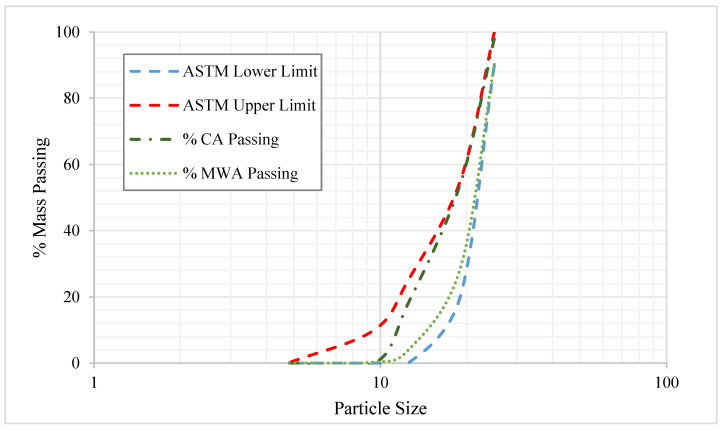
The particle size of MWA and standard aggregates is within the ASTM limits.

The marble waste aggregates (MWA) were obtained from a marble factory and further crushed and sieved to obtain the desired size of marble aggregates equivalent to the standard coarse aggregate size used in mix design [45]. The particle size distribution of MWA and a comparison with the standard coarse aggregate size are presented in Figure 1. 

The physical properties of MWA, which are relatable to the coarse aggregate utilized in the present study, are also given in Table 3.

**Figure 2:**
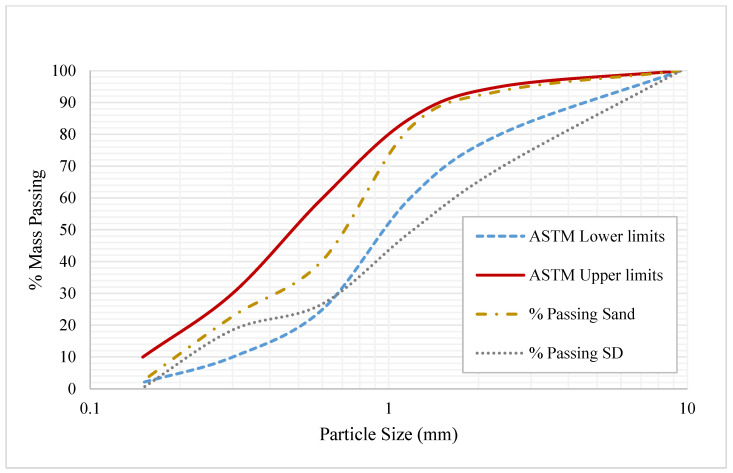
Particle size range of sand and SD.

Stone dust (SD) was procured from the aggregate crushing plant, which is the residue left after aggregate production in the crushing plant. The obtained stone dust was used as a replacement for sand in the mix design. The particle size of SD confirmed the standard size of sand, as shown in Figure 2. The water absorption and specific gravity of SD were determined as 1.48 and 2.61, respectively, with a fineness modulus of 3.35. 

### 2.2. Mix Design and Preparation of Specimens

For the experimental program, two approaches for mix design of concrete were tested. The ACI method of concrete mix design as well as conventional 1:2:4 mix design. The ACI method of mix design [46] recommends the relationship between average compressive strength (28-day) with water-to-cement ratio, free water content, cement content, as well as plastic densities, and quantities of aggregates. Since there is very less difference in bulk densities of coarse aggregates and marble aggregates as well as the fineness modulus of sand and stone dust. Some minute adjustments were carried out before casting the samples. The conventional 1:2:4 mix design, which proportions the cement, fine and coarse aggregates by weight, was used to ascertain the desired attributes of concrete in fresh and hardened states. From previous literature, it was also found that researchers, while finding replacement percentages of aggregates, not only employed the ACI mix design technique but also utilized conventional ratios of concrete 1:2:4, 1:1.5:3, etc., as well. By-weight mix design is somewhat of an empirical approach, but it is also confirmed with the ACI mix design method. Calculated values for unit volume are listed in Table 4. 

Standard practice for making and curing concrete sampling and casting of cylinders conforming to ASTM C192/C192M-14 [47] was carried out. Each mix design (1:2:4 by weight) had cylinders of 6″ (150 mm) diameter × 12″ length (300 mm), prisms/beams of 4″ (100 mm) × 4″ (100 mm) × 16″ (400 mm), and cubes of 6″ (150 mm) × 6″ (150 mm) × 6″ (150 mm). The water-cement ratio (w/c) of 0.5 was kept consistent for all mixes. A total of 189 samples were cast as per mix designs. Substitutions were achieved by utilizing 10%, 20%, and 30% marble waste aggregate as a replacement for coarse aggregate and 40%, 50%, and 60% substitution of stone dust in place of sand. The samples were designated as per the substitution percentages of aggregates. The sample designation was such that 10MWA means 10% MWA was substituted in place of standard coarse aggregate, 20MWA means 20% MWA was substituted in place of standard coarse aggregate, 30MWA means 30% MWA was substituted in place of standard coarse aggregate, 40SD means 40% SD was substituted in place of standard fine aggregate, 50SD means 50% SD was substituted in place of standard fine aggregate, and 60SD means 60% SD was substituted in place of standard fine aggregate. For each test and each category of substitution, at least three samples of cylinders and at least two samples of prisms/cubes were made. A total of 189 samples were initially cast, with designated categories. Then, to confirm the RSM model optimization experimentally, 63 samples were further cast. The cast samples remained in their respective molds for twenty-four hours, then were unmolded the next day and put in a water bath at ambient temperature. The samples were cured for the respective test requirements of 7 days and 28 days.

### 2.3. Workability and Absorption Tests

The properties of concrete in fresh form, i.e., workability tests such as the slump test confirming ASTM C143-78 [48]), the compaction factor test satisfying BS 1881: Part 103 [49], the density of fresh concrete satisfying ASTM C138/C138M-17a [50], and observation for bleeding water and segregation was performed. Comparisons of each test for control, marble waste aggregate substitution, and stone dust substitution in coarse and fine aggregate, respectively, are listed in succeeding sections. 

Slump for the control concrete specimen was selected at 2 inches (50 mm) and verified in the other two types of specimen (MWA and SD samples) as well. To ascertain the workability of fresh concrete, another method is the compaction factor test. The option slump test is suitable for more workable concrete, whereas the compaction factor test is suitable for less workable concrete. The compaction factor workability test, comprising of three steps, dropping the concrete from 8 inches (200 mm) in two hoppers, then collecting it in a cylinder, and yielding the potential energy as the internal work done on the concrete. This free-dropped concrete weight is known as the weight of concrete with partial compaction due to potential energy (height). Then the cylinder is again filled with fresh concrete and either vibrated externally or compacted in three layers utilizing a tamping rod, hence getting standard compaction. This compacted concrete cylinder weight is the weight of fully compacted concrete. The relationship in terms of compaction factor is:

$$\mathrm{Compacting}\;\mathrm{factor}=\frac{\mathrm{Partially}\;\mathrm{compacted}\;\mathrm{cylinder}\;\mathrm{weight}}{\mathrm{Fully}\;\mathrm{compacted}\;\mathrm{cylinder}\;\mathrm{weight}}$$

For the absorption test, the cast samples were first submerged for a period of 24 h at room temperature (25 to 30 °C) in clean water, then weighed in surface dry condition. After that, the samples in wet condition were put in the oven for 24 h and weighed once again, in compliance with ASTM C642-21 [51]. The percentage absorption is calculated as the following formula:

$$Absorption=\frac{100\times \left(Wet\;Sample\;weight-Dry\;Sample\;weight\right)}{Dry\;Sample\;weight}$$

### 2.4. Concrete Strength Tests: Compressive Strength, Flexural Strength, and Split Tensile Strength Test

The compressive strength test was performed in accordance with ASTM C39/C39M-21 [52], for measuring the capacity of any material that it can sustain before fracturing by applying the maximum amount of compressive load. The concrete test samples were cylinders, beams or prisms, and cubes. After curing for 7 days and 28 days, the specimens were tested as per ASTM standards. The specimens were tested in a compression testing machine by applying gradual loads as per the strain rate in Table 5. The average strength of the tested sample was selected. 

An ASTM C78/C78M-22 [53] third point loading test was performed to measure the flexural strength of concrete specimens. This test measures the resistance of unreinforced beams or slabs to failure due to bending moments caused by the applied load. It is conducted by loading a 4″ × 4″ (100 mm × 100 mm) concrete prism that has a length of more than three times its depth. The modulus of rupture (M_R_) is also the measure of flexural strength. Usually, it is 10 to 20% of the compressive strength, depending on the aspect ratio of the specimen’s shape and the volume of coarse aggregate in the mix. 

ASTM C496-96 [54] was performed to ascertain the splitting tensile strength of cylindrical concrete specimens by placing the cylinder specimen horizontally in the compression testing machine. The depth of the cylinder was 6″ (150 mm) and the length of the cylinder was 12″ (300 mm). 

### 2.5. Durability Test: Acid Attack Resistance 

ASTM C1898-20 [55] was performed to ascertain the chemical resistance of the concrete specimens under the anticipated service environment. The specimens were cast in a 4″ (100 mm) × 4″ (100 mm) cube and cured for 7 days. Then, these specimens were left in dry condition for 3 days to attain a constant weight. Then, specimens were weighed and put in a 5% solution of sulphuric acid (H_2_SO_4_) for 24 h, and after being removed from the solution and washed, the change in various parameters of a specimen, such as weight and appearance of the specimen, the test medium’s appearance, as well as the specimen’s compressive strength, were evaluated. 

### 2.6. Application of RSM

For experimental design, modeling, prediction, and optimization, and establishing a relationship between experimental and predicted data, a user-friendly statistical tool is response surface methodology (RSM) [8,26,56,57,58,59]. Statistical and mathematical features are combined in RSM to effectively design the experiment and establish a relationship between variables and responses [60,61,62]. In the current study, Design Expert^®^ software was used, which is commercially available. Among other methods, central composite design (CCD) is the most commonly used RSM design method in the construction industry [63] and therefore also adopted in this study. 

In this study, MWA and SD were considered as input factors (independent variables), and the responses (dependent variables) were selected as strength properties such as the compressive strength (CS), flexural strength (FS), and split tensile strength (TS) of concrete specimens. The aim was to see the effect of MWA and SD on the prediction of CS, FS, and TS.

## 3. Results and Discussion

### 3.1. Effect of MWA and SD Substitution on Physical and Durability Properties

#### 3.1.1. Workability

**Figure 3:**
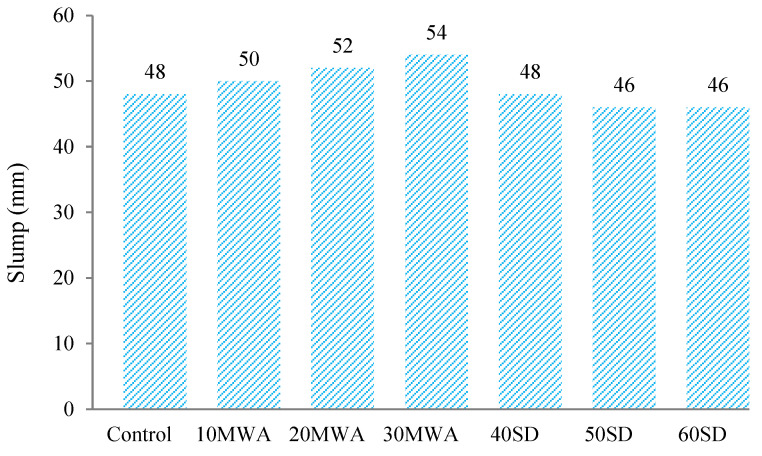
Slump values for MWA and SD based concrete.

The slump test measures the workability of concrete. The desired slump value for the control specimen was 50 mm. All results were within the range of 50 ± 5 mm (Figure 3). With the addition of MWA, slump increases are attributed to the smooth surface and less absorption of MWA than normally used coarse aggregate. These results were validated by past researchers as well [17]. On the other hand, substation of SD causes decreases in slump value, which indicates that SD has finer particles in contrast to sand.

**Figure 4:**
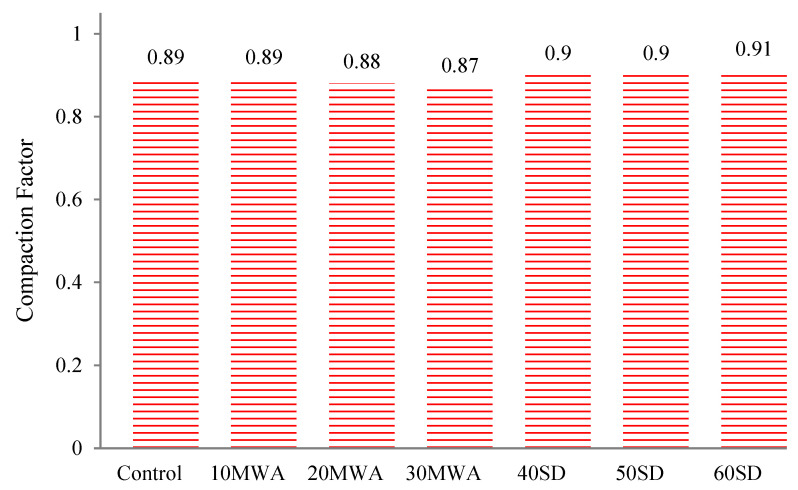
Compaction Factor Test.

Workability can also be assessed by the compaction factor test, as shown in Figure 4. The desired value for the control specimen was 0.92. All workability test results were within the range of 0.90 ± 0.05, which confirms comparison consistency between the examined properties of the concrete samples. With the addition of marble waste aggregate (MWA), the compaction factor decreases as the ratio rises. This can be attributed to the smooth surface and less absorption of MWA than normally used coarse aggregate. While the inclusion of stone dust (SD), the compaction factor increases as the percentage of SD increases. This can be attributed to finer particles of SD as compared to sand. The results are in close agreement with previous research findings [37].

#### 3.1.2. Absorption Test

**Figure 5:**
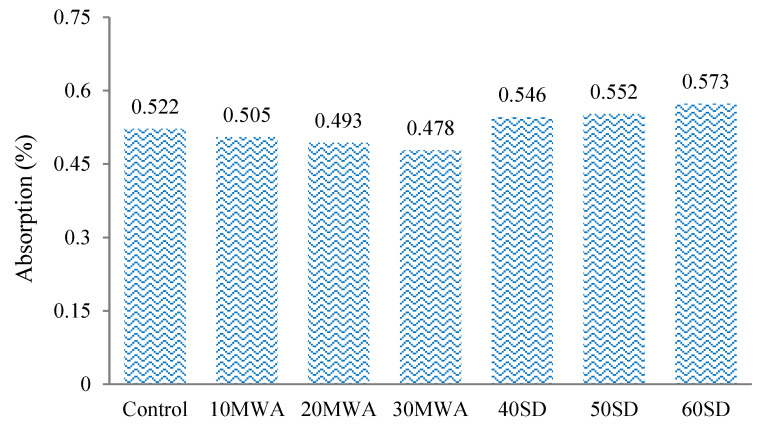
Absorption Percentage of specimens containing MWA and SD.

The average absorption percentages (Figure 5) of samples indicated that the absorption of samples containing MWA was less (3.25% to 5.55%) than the control sample, which may be attributed to the more polished surfaces of MWA than the normal aggregate, whereas the absorption of samples containing SD was more (4.6% to 9.77%) than the control sample, which may be attributed to finer particles (exhibiting more surface area) present in SD than the normal sand [64,65]. 

#### 3.1.3. Acid Attack Resistance 

**Figure 6:**
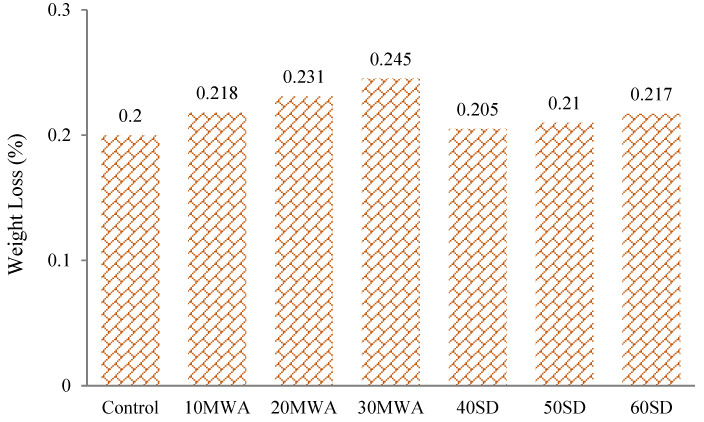
Weight loss of specimen after exposure to acid attack.

ASTM C1898-20 was performed to ascertain the chemical resistance of the concrete specimens under the anticipated service environment. The solutions became ashy after one day of submersion of the samples. There was some grainy appearance after the removal of samples from the acid, and this appearance was rougher in specimens containing MWA as compared to the control sample, whereas the appearance was unchanged in the case of SD samples (Figure 6). These findings may be ascribed to the more reactive nature of MWA with H_2_SO_4_ than the normal aggregate and finer particles of SD in the respective samples [64]. 

### 3.2. Effect of MWA and SD on Strength Properties and Statistical Modeling

Statistical analysis in RSM was performed and validated via ANOVA, fit statistics, and proposed models. Based on higher R^2^, quadratic models were suggested for compressive strength, flexural strength, and tensile strength as shown in Table 6, Table 7 and Table 8, respectively. The generalized quadratic polynomial Equation (1) is used to predict the response value (i.e., compressive strength, flexural strength, and tensile strength).

(1) $$Y=C+A_{1}\left(X_{1}\right)+A_{2}\left(X_{2}\right)+A_{3}\left(X_{1}*X_{2}\right)+A_{4}\left(X_{1}{}^{2}\right)+A_{5}\left(X_{2}{}^{2}\right).$$

where Y = predicted response, X_1_ and X_2_ = independent variables (MWA and SD), C = interception constant, A_1_ and A_2_ = linear coefficients, A_3_ = interaction coefficient, and A_4_ and A_5_ = quadratic coefficients.

#### 3.2.1. Effect on Compressive Strength and Statistical Analysis

Table 6 shows the ANOVA and fit statistics of 7-day and 28-day compressive strength. ANOVA was used to evaluate the significance of independent factors and their effects on responses. The validation and suitability of regression models for compressive strength were performed by using the coefficient of determination (R^2^), adjusted R^2^, and predicted R^2^, as shown in Table 6. Based on the fit summary and ANOVA, RSM suggested quadratic models for compressive strength at 7 days and 28 days. The higher values of coefficient of determination (R^2^ ≥ 0.90) and higher model F-value (>40) indicate that the proposed models are significant and there is a substantial relationship between factors and corresponding responses. The *p*-value of less than 0.05 also shows that the proposed model terms are significant with more than a 95% confidence level. Similarly, the numerical difference between adjusted R^2^ and predicted R^2^ of less than 0.2 (Table 6) also indicates close agreement between predicted and experimental results. 

**Figure 7:**
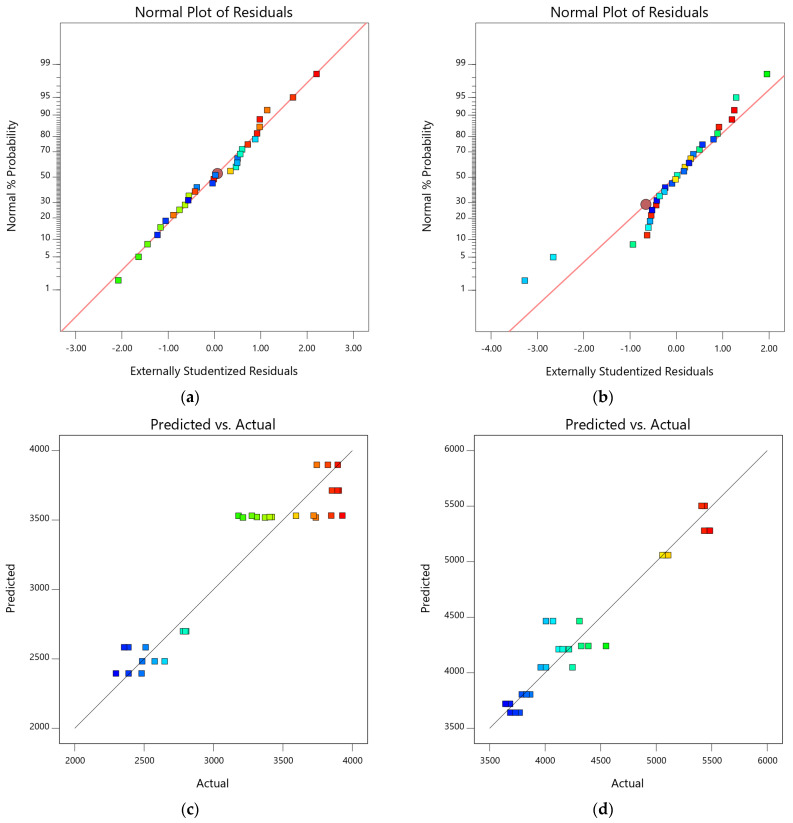

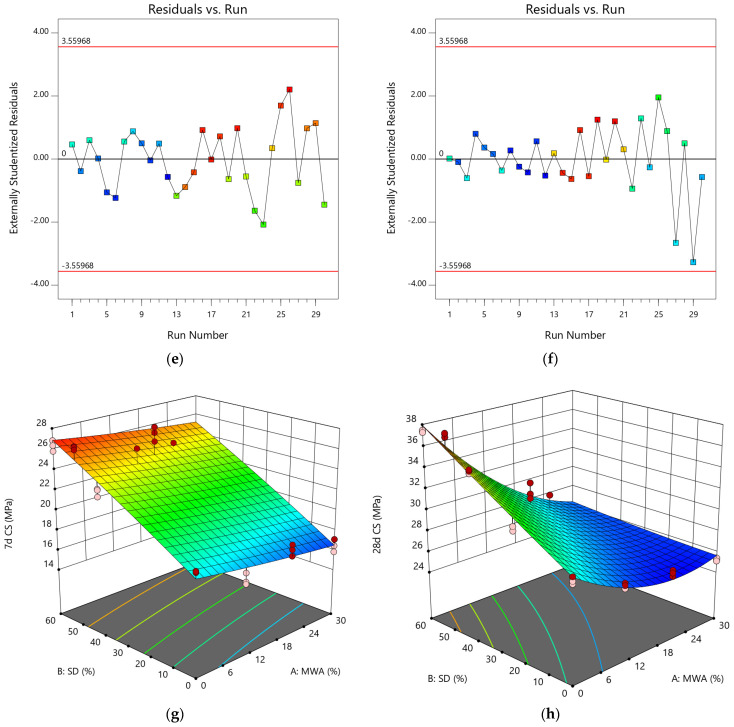
Diagnostic and 3D plots of compressive strengths at 7 days and 28 days. (**a**) 7-day compressive strength, (**b**) 28-day compressive strength, (**c**) 7-day compressive strength, (**d**) 28-day compressive strength, (**e**) 7-day compressive strength, (**f**) 28-day compressive strength, (**g**) 7-day compressive strength, (**h**) 28-day compressive strength.

In addition to model validation and fit statistics, the adequacy and normal distribution of data can also be verified graphically by diagnostic plots. The diagnostic plots, such as normal plots of residual, predicted vs. actual plots, and residual vs. run plots, can be used in evaluating the accuracy of regression analysis [66]. The normal plots of residual for both 7-day and 28-day compressive strength are shown in Figure 7a,b. It can be observed from the plots that the relationship between the normal percentage of probability and the extremely studentized residual is satisfactory because almost all the points are closely located along the straight line for both responses. It indicates that the residual values, which are the difference between experimental and predicted responses, exhibit a normal distribution [67]. The relationship between predicted and actual data points for 7-day and 28-day compressive strength is shown in Figure 7c,d. The distribution of data points is spread relatively close to the equality line, which indicates satisfactory fitting precision of the models and adequate agreement between experimental and predicted results. Similarly, Figure 7e,f represent the plot of residuals with respect to predicted responses. The data scattering within the top and bottom red boundaries and the sinusoidal distribution of data points corresponding to run number indicate acceptable accuracy of model predictability. These diagnostic plots validate that the developed compressive strength models are applicable to predict the 7-day and 28-day compressive models corresponding to MWA and SD content [60,61,62]. Moreover, 3D graphs (Figure 7g,h) can also be used to establish a relationship between factors (SD and MWA) and responses (CS). Compressive strength at 7 days and 28 days of curing showed a decline with increasing MWA substitution. However, the substitution of SD showed an increase in compressive strength. 

#### 3.2.2. Effect on Flexural Strength and Statistical Analysis

Table 7 shows the ANOVA and fit statistics of 7-day and 28-day flexural strength. ANOVA was used to evaluate the significance of independent factors and their effects on responses. The validation and suitability of regression models for flexural strength were performed by using the coefficient of determination (R^2^), adjusted R^2,^ and predicted R^2^, as shown in Table 7. Based on the fit summary and ANOVA, RSM suggested quadratic models for flexural strength at 7 days and 28 days. The higher values of coefficient of determination (R^2^ ≥ 0.90) and higher model F-value (>40) indicate that the proposed models are significant and there is a substantial relationship between factors and corresponding responses. The *p*-value of less than 0.05 also shows that the proposed model terms are significant with more than a 95% confidence level. Similarly, the numerical difference between adjusted R^2^ and predicted R^2^ of less than 0.2 (Table 7) also indicates close agreement between predicted and experimental results for 7-day and 28-day flexural strength. 

**Figure 8:**
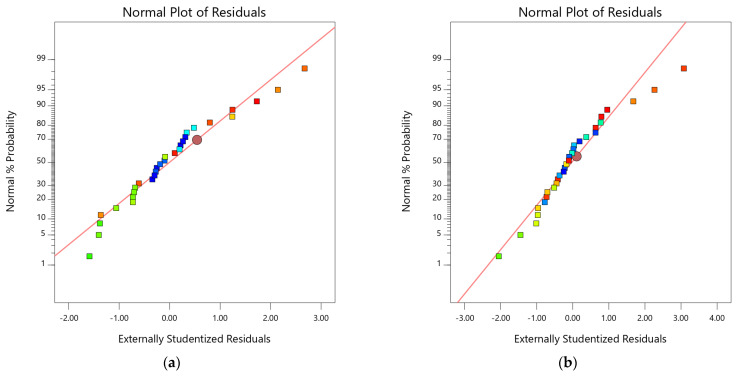

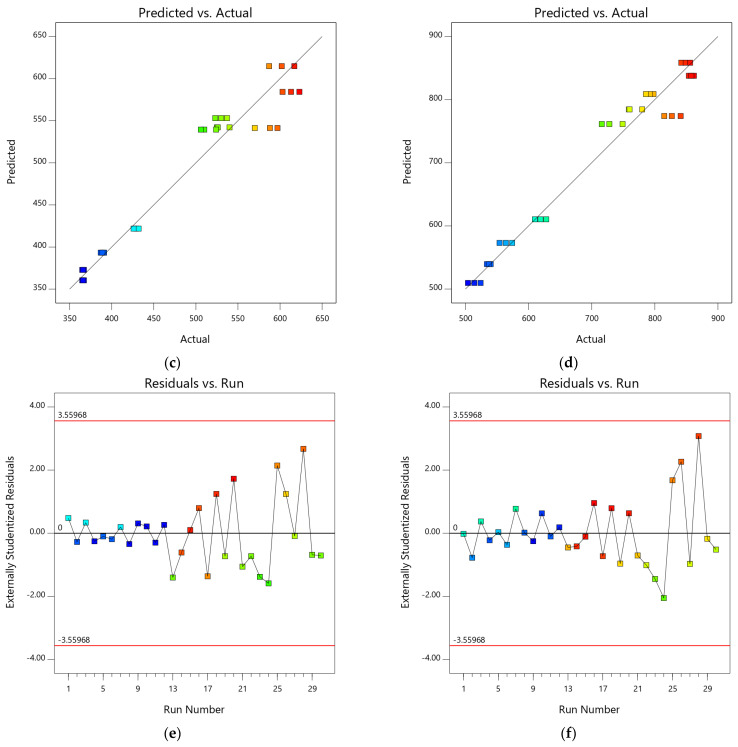

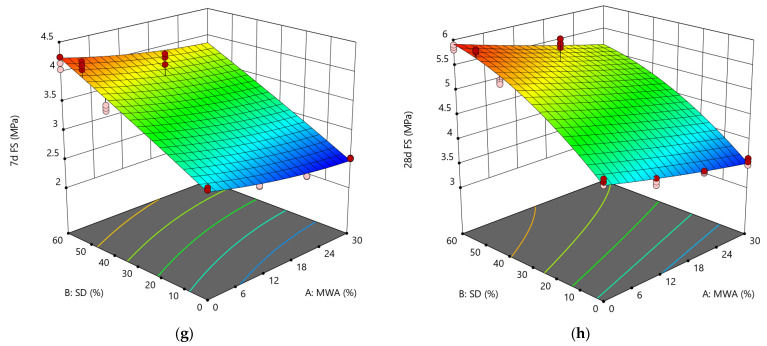
Diagnostic and 3D plots of 7-day and 28-day flexural strength. (**a**) 7-day flexural strength, (**b**) 28-day flexural strength, (**c**) 7-day flexural strength, (**d**) 28-day flexural strength, (**e**) 7-day flexural strength, (**f**) 28-day flexural strength, (**g**) 7-day flexural strength, (**h**) 28-day flexural strength.

In addition to model validation and fit statistics, the adequacy and normal distribution of data can also be verified graphically by diagnostic plots. The diagnostic plots, such as normal plots of residual, predicted vs. actual plots, and residual vs. run plots, can be used in evaluating the accuracy of regression analysis [66]. The normal plots of residual for both 7-day and 28-day flexural strength are shown in Figure 8a,b. It can be observed from the plots that the relationship between the normal percentage of probability and the extremely studentized residual is satisfactory because almost all the points are closely located along the straight line for both responses. It indicates that the residual values, which are the difference between experimental and predicted responses, exhibit a normal distribution [67]. The relationship between predicted and actual data points of 7-day and 28-day flexural strength is shown in Figure 8c,d. The distribution of data points is spread relatively close to the equality line, which indicates satisfactory fitting precision of the models and adequate agreement between experimental and predicted results. Similarly, Figure 8e,f represent the plot of residuals with respect to predicted responses. The data scattering within the top and bottom red boundaries and the sinusoidal distribution of data points corresponding to run number indicate acceptable accuracy of model predictability. These diagnostic plots validate that the developed flexural strength models are applicable to predict the 7-day and 28-day flexural models corresponding to MWA and SD content [60,61,62]. Moreover, 3D graphs (Figure 8g,h) can also be used to establish a relationship between factors (SD and MWA) and responses (FS). Flexural strength at 7 days and 28 days of curing showed a decline with increasing MWA substation. However, the substitution of SD showed an increase in flexural strength.

#### 3.2.3. Effect on Tensile Strength and Statistical Analysis

Table 8 shows the ANOVA and fit statistics of 7-day and 28-day tensile strength. ANOVA was used to evaluate the significance of independent factors and their effects on responses. The validation and suitability of regression models for flexural strength were performed by using the coefficient of determination (R^2^), adjusted R^2,^ and predicted R^2^, as shown in Table 8. Based on the fit summary and ANOVA, RSM suggested quadratic models for tensile strength at 7 days and 28 days. The higher values of coefficient of determination (R^2^ = 0.89) and higher model F-value (>40) indicate that the proposed models are significant and there is a substantial relationship between factors and corresponding responses. The *p*-value of less than 0.05 also shows that the proposed model terms are significant with more than a 95% confidence level. Similarly, the numerical difference between adjusted R^2^ and predicted R^2^ of less than 0.2 (Table 8) also indicates close agreement between predicted and experimental results for 7-day and 28-day tensile strength. 

**Figure 9:**
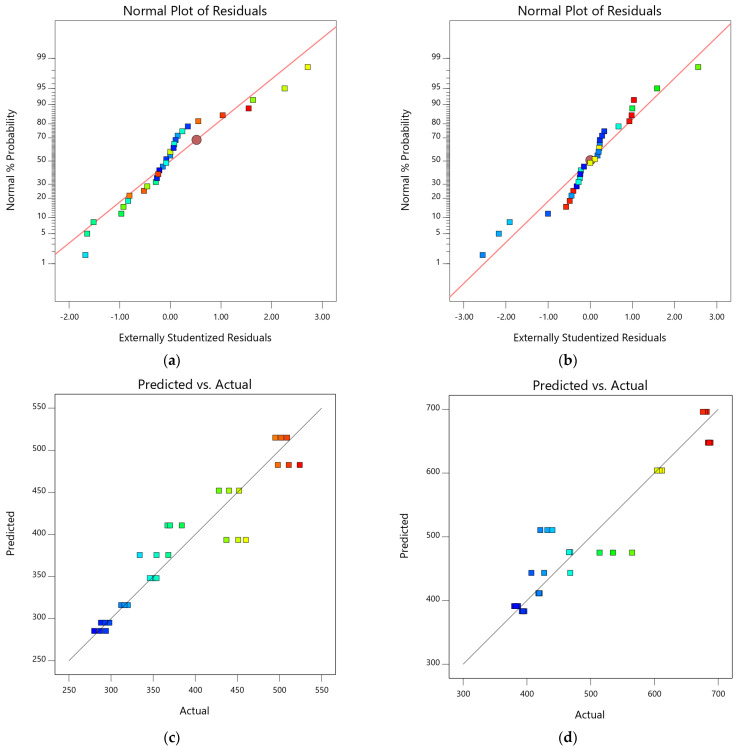

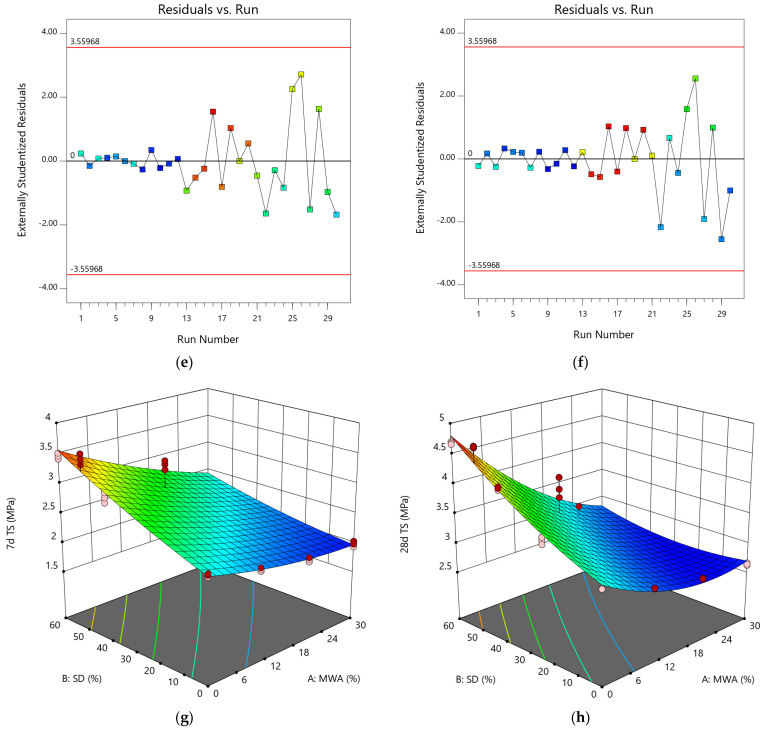
Diagnostic and 3D plots of 7-day and 28-day split tensile strength. (**a**) 7-day split tensile strength, (**b**) 28-day split tensile strength, (**c**) 7-day split tensile strength, (**d**) 28-day split tensile strength, (**e**) 7-day split tensile strength, (**f**) 28-day split tensile strength, (**g**) 7-day split tensile strength, (**h**) 28-day split tensile strength.

In addition to model validation and fit statistics, the adequacy and normal distribution of data can also be verified graphically by diagnostic plots. The diagnostic plots, such as normal plots of residual, predicted vs actual plots, and residual vs run plots, can be used in evaluating the accuracy of regression analysis [66]. The normal plots of residual for both 7-day and 28-day tensile strength are shown in Figure 9a,b. It can be observed from the plots that the relationship between the normal percentage of probability and the extremely studentized residual is satisfactory because almost all the points are closely located along the straight line for both responses. It indicates that the residual values, which are the difference between experimental and predicted responses, exhibit a normal distribution [67]. The relationship between predicted and actual data points for 7-day and 28-day tensile strength is shown in Figure 9c,d. The distribution of data points is spread relatively close to the equality line, which indicates a satisfactory fitting precision of the models and adequate agreement between experimental and predicted results. Similarly, Figure 9e,f represent the plot of residuals with respect to predicted responses. The data scattering within the top and bottom red boundaries and the sinusoidal distribution of data points corresponding to run number indicate acceptable accuracy of model predictability. These diagnostic plots validate that the developed tensile strength models are applicable to predict the 7-day and 28-day flexural models corresponding to MWA and SD content [60,61,62]. Moreover, 3D graphs (Figure 9g,h) can also be used to establish a relationship between factors (SD and MWA) and responses (TS). Tensile strength at 7-day and 28-day curing showed a decline with increasing MWA substitution. However, the substitution of SD showed an increase in tensile strength.

### 3.3. Prediction of Strength Properties 

Interestingly, RSM also gives the coefficient of modeled terms for predicting the response properties, in this case, compressive strength, flexural strength, and tensile strength of concrete containing MWA and SD as substitutes. The generalized quadratic polynomial Equation (1) can be modified by inserting the constant and coefficient of model terms (as shown in Table 9). These equations can be used to predict the corresponding strength properties of concrete containing MWA and SD as substitutes. 

Based on the above coefficients of equations (Table 9), the following prediction equations were generated using generalized Equation (1). The predicted values of the responses in Equations (2)–(7) are in MPa.

(2) $$CS_{7d}=18.61-0.08\left(\mathrm{MWA}\right)+0.15\left(\mathrm{SD}\right)+0.0346\left(\mathrm{MWA}\times \mathrm{SD}\right)+0.070\left({\mathrm{MWA}}^{2}\right)-0.0297\left({\mathrm{SD}}^{2}\right)$$

(3) $$CS_{28d}=29.04-0.36\left(\mathrm{MWA}\right)+0.14\left(\mathrm{SD}\right)-0.6585\left(\mathrm{MWA}\times \mathrm{SD}\right)+1.215\left({\mathrm{MWA}}^{2}\right)+0.017\left({\mathrm{SD}}^{2}\right)$$

(4) $$FS_{7d}=2.91-0.02\left(\mathrm{MWA}\right)+0.02\left(\mathrm{SD}\right)+0.0041\left(\mathrm{MWA}\times \mathrm{SD}\right)+0.0402\left({\mathrm{MWA}}^{2}\right)-0.0032\left({\mathrm{SD}}^{2}\right)$$

(5) $$FS_{25d}=4.21-0.027\left(\mathrm{MWA}\right)+0.05\left(\mathrm{SD}\right)-0.0055\left(\mathrm{MWA}\times \mathrm{SD}\right)+0.0187\left({\mathrm{MWA}}^{2}\right)-0.0412\left({\mathrm{SD}}^{2}\right)$$

(6) $$TS_{7d}=2.4-0.026\left(\mathrm{MWA}\right)+0.015\left(\mathrm{SD}\right)-0.0541\left(\mathrm{MWA}\times \mathrm{SD}\right)+0.0555\left({\mathrm{MWA}}^{2}\right)+0.0091\left({\mathrm{SD}}^{2}\right)$$

(7) $$TS_{28d}=3.28-0.06\left(\mathrm{MWA}\right)+0.015\left(\mathrm{SD}\right)-0.1117\left(\mathrm{MWA}\times \mathrm{SD}\right)+0.1811\left({\mathrm{MWA}}^{2}\right)+0.0233\left({\mathrm{SD}}^{2}\right)$$

### 3.4. Optimization and Validation of Results

The multi-objective optimization technique was performed while considering all factors (MWA and SD dosages) and responses (compressive, flexural, and tensile strengths at 7 days and 28 days). The results (Table 10) indicated that 15% replacement of MWA and 50% SD gives maximum strength properties compared with the control and all other combinations. The predicted results from the optimum combination were also validated through additional experiments. The error of less than 5% indicates that the precited results are in good agreement with experimental data [61,62]. 

### 3.5. Cost Effect of Utilizing the MWA and SD

The marble waste aggregate and stone dust were both obtained from the dump sites of the respective factories free of charge. This saved an overall 15–18% cost for the optimized proportion in the production of concrete compared to the standard aggregates.

## 4. Conclusions

The partial substitution of marble waste aggregate (MWA) and stone dust (SD) was used to replace natural coarse and fine aggregates, respectively, in developing normal strength concrete. The concrete specimens were subjected to physical, durability, and strength tests. The following conclusion is drawn from this study.

1. With the addition of MWA, workability increases (up to slump +12% and compaction factor −2%) attributed to the smooth surface and less absorption of MWA than normally used coarse aggregate. On the other hand, substation of SD causes decreases in workability (up to slump −4% and compaction factor +2%), which is attributed to the finer particles of SD as compared to sand. Similarly, up to 8.4% less absorption in the MWA specimen and 9.8% more absorption than the control specimen were noted.
2. Resistance to chemicals (acid attack) was less prominent in MWA (22% loss of material) and SD (8.5% loss of material) as compared to the standard control specimen.
3. With a lower crushing value of MWA of 19.98%, as opposed to 16.98% of standard coarse aggregate, a relatively smoother surface of MWA than standard coarse aggregate, and less water absorption (paste adhesion), the bond of MWA was weaker than standard coarse aggregate, as obviously visible in the fractured samples.
4. Due to better packing of a wide range of particle sizes of SD, i.e., a wider range of particle gradation, and relatively higher specific gravity improved the density of concrete to some extent. In general, the density relates to the durability of concrete in later stages of service life. However, due to the likely increase of more uniform-sized particles beyond optimum content, there was no clear increase observed in strength parameters.
5. Compressive strength was observed to be slightly lower (14% at 7 days and 12% at 28 days) with 30% MWA substitution but significantly enhanced with 50% SD substitutions (39% at 7 days and 31% at 28 days) individually, as compared to the control sample. However, the strength enhancement declines again with additional SD content.
6. Flexural strength was observed to be slightly lower (15% at 7 days and 17% at 28 days) up to 30% MWA substitution but significantly enhanced (42% at 7 days and 39% at 28 days) up to 50% SD substitutions individually, as compared to the control sample.
7. Split tensile strength was observed to be slightly lower (18% at 7 days and 18% at 28 days) up to 30% MWA substitution but significantly enhanced (46% at 7 days and 47% at 28 days) up to 50% SD substitutions individually, as compared to the control sample.
8. For establishing the combined effect of MWA and SD, RSM was applied, and based on contour plots, three sets of MWA and SD substitutions were obtained for comparing compressive strength with the target compressive strength of 3400 psi and 4200 psi at 7 days and 28 days, respectively. The ensuing compressive strength was 3440 psi (7-day) and 4129 psi (28-day) with the 10MWA-45SD combination, 3832 psi (7-day) and 4421 psi (28-day) with the 15MWA-50SD combination, and 3350 psi (7-day) and 4071 psi (28-day) with the 20MWA-50SD combination.
9. A significant relationship between RSM predicted data and experimental results was achieved for compressive strength, flexural strength, and tensile strength. The proposed quadratic models are well fitted due to a higher R^2^ (>0.80) and a lower *p*-value (<0.05), and hence the derived equations can be used to predict compressive strength, flexural strength, and tensile strength.
10. MWA of 15% and SD of 50% replacements were obtained as optimized dosages to replace coarse aggregates and fine aggregates, respectively. The predicted optimized data was validated by additional experiments with an error of less than 5%.
11. An encouraging aspect of the research showed that at an early age, the strength development of the optimized mix is significant. It may be attributed to the rougher texture of SD particles and less absorption of MWA, thus leaving water available for hydration as the combined effect of both substitutes created the opportunity for a stronger bond at the initial phases of hydration. It was observed that approximately 70% of the ultimate strength was achieved in the initial 7 days when cured at a curing temperature of 27 °C ± 5 °C.
12. Re-utilization of waste materials and byproducts (such as marble waste aggregate and stone dust), while reducing the consumption of natural aggregates, will lead to achieving sustainability and producing green concrete.

## Figures and Tables

**Table 1:** Properties of cement.

Cement Properties	Values Obtained
Normal Consistency	32%
Initial Setting Time	47 min
Final Setting Time	287 min
Fineness	4.5%
Specific Gravity	3.15

**Table 2:** Physical properties of coarse and fine aggregates.

Physical Properties	Fine Aggregates	Coarse Aggregates
Specific Gravity	2.6	2.83
Water Absorption (%)	1.28	3.4
Fineness Modulus	2.5	-
Bulk Density	-	1570 kg/m^3^ (98 lb/ft^3^)
Crushing Value (%)	-	16.98

**Table 3:** Physical properties of MWA.

Physical Properties	MWA	Coarse Aggregate
Specific Gravity	2.72	2.83
Water Absorption (%)	3.15	3.4
Bulk Density (kg/m^3^)	1520 kg/m^3^ (95 lb/ft^3^)	1570 kg/m^3^ (98 lb/ft^3^)
Crushing Value	19.98	16.98

**Table 4:** Mix design quantities of standard constituents of control specimen.

Input Parameters	By-Weight Values FPS (SI) Units
Required Cylinder Compressive Strength	4500 psi (31 MPa)
Required Slump	2 inch (50 mm)
Nominal maximum aggregate size	3/4 inch (20 mm)
w/c ratio of mix	0.5
Water quantity	270 lb/yd^3^ (160 kg/m^3^)
Quantity of Cement	540 lb/yd^3^ (320 kg/m^3^)
Plastic Density	4096 lb/yd^3^ (2430 kg/m^3^)
Aggregate Contents [Coarse + Fine]	3288 lb/yd^3^ (1950 kg/m^3^)
Fineness modulus of sand	2.5
Bulk volume of	0.67
Quantity of Coarse Aggregate	2192 lb/yd^3^ (1300 kg/m^3^)
Quantity of Fine Aggregate	1096 lb/yd^3^ (650 kg/m^3^)

**Table 5:** Loading and Strain rates for Compression testing of the specimen.

Sample	Min Load	Max Load	Min Strain Rate	Max Strain Rate
For 6″ (150 mm) × 6″ (150 mm) cube	63 Kips (280 KN)	108 Kips (480 KN)	8.1 Kips/min (36 KN/min)	13.5 Kips/min (60 KN/min)
For 6″ (150 mm) × 12″ (300 mm) Cylinder	50 Kips (220 KN)	83 Kips (370 KN)	6.8 Kips/min (30 KN/min)	14.6 Kips/min (65 KN/min)

**Table 6:** ANOVA and fit statistics of compressive strength.

Responses	7-Day CS MPa (psi)	28-Day CS MPa (psi)
Standard Deviation	1.41 (204.90)	1.21 (175.92)
Mean	21.98 (3187.43)	30.32 (4397.60)
R^2^	0.90	0.94
Adjusted R^2^	0.88	0.93
Predicted R^2^	0.85	0.92
Adequate Precision	16.39	23.66
Model F-value	42.37	77.58
Model *p*-value	<0.0001	<0.0001
Model Remarks	Significant	Significant
Proposed Model	Quadratic	Quadratic

**Table 7:** ANOVA and fit statistics of flexural strength.

Responses	7-Day FS MPa (psi)	28-Day FS MPa (psi)
Standard Deviation	0.17 (24.72)	0.18 (26.78)
Mean	3.39 (492.37)	4.87 (705.80)
R^2^	0.94	0.96
Adjusted R^2^	0.93	0.96
Predicted R^2^	0.92	0.95
Adequate Precision	23.01	29.12
Model F-value	79.20	132.23
Model *p*-value	<0.0001	<0.0001
Model Remarks	Significant	Significant
Proposed Model	Quadratic	Quadratic

**Table 8:** ANOVA and fit statistics of tensile strength.

Responses	7-Day TS MPa (psi)	28-Day TS MPa (psi)
Standard Deviation	0.20 (29.21)	0.28 (41.29)
Mean	2.67 (387.40)	3.47 (503.90)
R^2^	0.89	0.89
Adjusted R^2^	0.87	0.87
Predicted R^2^	0.85	0.85
Adequate Precision	17.57	16.95
Model F-value	41.22	41.29
Model *p*-value	<0.0001	<0.0001
Model Remarks	Significant	Significant
Proposed Model	Quadratic	Quadratic

**Table 9:** Coefficients of the equation to predict responses.

Coefficients of Equation		Responses					
		Compressive Strength at 7-Day MPa (psi)	Compressive Strength at 28-Day	Flexural Strength at 7-Day	Flexural Strength at 28-Day	Tensile Strength at 7-Day	Tensile Strength at 28-Day
Constant	C	18.61 (+2699.1)	29.04 (+4212.6)	2.91 (+421.9)	4.21 (+610.4)	2.4 (+348.1)	3.28 (+475.8)
MWA	A_1_	−0.08 (−12.3)	−0.36 (−52.9)	−0.02 (−3.3)	−0.027 (−3.9)	−0.026 (−3.8)	−0.06 (−8.2)
SD	A_2_	0.15 (+21.7)	0.14 (+20.5)	0.02 (+3.4)	0.05 (+6.6)	0.015 (+2.3)	0.015 (+2.3)
MWA × SD	A_3_	+0.0346	−0.6585	+0.0041	−0.0055	−0.0541	−0.1117
MWA^2^	A_4_	+0.0700	+1.2150	+0.0402	+0.0187	+0.0555	+0.1811
SD^2^	A_5_	−0.0297	+0.0170	−0.0032	−0.0412	+0.0091	+0.0233

**Table 10:** Optimization of factor and experimental validation.

	MWA (%)	SD (%)	7-Day CS MPa (psi)	28-Day CS MPa (psi)	7-Day FS MPa (psi)	28-Day FS MPa (psi)	7-Day TS MPa (psi)	28-Day TS MPa (psi)
RSM results	15	50	24.62 (3571.01)	29.41 (4265.65)	3.78 (547.65)	5.37 (779.07)	2.75 (398.13)	3.32 (480.92)
Additional Experimental data	15	50	25.37 (3679.2)	30.58 (4435.42)	3.91 (567.36)	5.14 (745.14)	2.67 (387.98)	3.26 (473.24)
Error (%)	--	--	3	4	4	4	3	2
